# Ternary WD40 Repeat-Containing Protein Complexes: Evolution, Composition and Roles in Plant Immunity

**DOI:** 10.3389/fpls.2015.01108

**Published:** 2016-01-07

**Authors:** Jimi C. Miller, William R. Chezem, Nicole K. Clay

**Affiliations:** ^1^Department of Molecular Biophysics and Biochemistry, Yale UniversityNew Haven, CT, USA; ^2^Department of Molecular, Cellular and Developmental Biology, Yale UniversityNew Haven, CT, USA

**Keywords:** heterotrimeric G-protein, MYB-bHLH-WDR complex, TTG1, Gβ, plant innate immunity, RACK1, flavonoid metabolism

## Abstract

Plants, like mammals, rely on their innate immune system to perceive and discriminate among the majority of their microbial pathogens. Unlike mammals, plants respond to this molecular dialog by unleashing a complex chemical arsenal of defense metabolites to resist or evade pathogen infection. In basal or non-host resistance, plants utilize signal transduction pathways to detect “non-self,” “damaged-self,” and “altered-self”- associated molecular patterns and translate these “danger” signals into largely inducible chemical defenses. The WD40 repeat (WDR)-containing proteins Gβ and TTG1 are constituents of two independent ternary protein complexes functioning at opposite ends of a plant immune signaling pathway. They are also encoded by single-copy genes that are ubiquitous in higher plants, implying the limited diversity and functional conservation of their respective complexes. In this review, we summarize what is currently known about the evolutionary history of these WDR-containing ternary complexes, their repertoire and combinatorial interactions, and their downstream effectors and pathways in plant defense.

## Introduction

WD40 repeat (WDR)-containing proteins are prevalent in eukaryotes, but rarely present in prokaryotes (Janda et al., 1996; Stirnimann et al., 2010). Plant genomes typically encode more than 200 putative WDR-containing proteins (van Nocker and Ludwig, 2003; Ouyang et al., 2012), which is slightly less than the human genome (349; Letunic et al., 2014). The basic function of WDR- containing proteins is to serve as rigid scaffolds for protein–protein and protein-DNA interactions. WDR-containing proteins are involved in fundamental mechanisms such as signal transduction, chromatin modification and transcriptional regulation. They are also involved in a wide variety of plant processes, including cell division, meristem organization, light signaling, floral development, secondary metabolism, and innate immunity (Smith et al., 1999; van Nocker and Ludwig, 2003; Perfus-Barbeoch et al., 2004).

Plants, unlike mammals, lack mobile defender cells and an adaptive immune system. Instead, they rely on the innate immunity of each cell, systemic peptide and chemical signals emanating from infection sites, and preformed and inducible chemical defenses at infection sites to ward off invading pathogens (Dixon, 2001; Jones and Dangl, 2006; Zipfel, 2014). Plants, like mammals, have a multi-tiered pathogen-detection system. The first layer is evolutionarily more ancient and involves the cell-surface perception of conserved microbial or “non self” molecular signatures known as microbe-/pathogen-associated molecular patterns (or MAMPs/PAMPs) and pathogen-generated “damaged/altered-self” molecular signatures known as damage-associated molecular patterns (or DAMPs). These “danger” signals are recognized by pattern recognition receptors (or PRRs), which in plants are plasma membrane-localized receptor-like proteins (RLPs) or receptor-like kinases (RLKs). MAMPs, *inter alia*, are also thought to be the molecular determinants of induced systemic resistance (ISR) that is activated by beneficial plant-microbe interactions in the roots (Van Wees et al., 1997; Meziane et al., 2005; Bakker et al., 2007). The second layer of immunity involves the cytosolic perception of pathogen-specific effector proteins by intracellular nucleotide binding leucine-rich repeat (or NB-LRR)-containing resistance proteins to trigger programmed cell death at infection sites and, in many cases, systemic acquired resistance in the host plant (Jones and Dangl, 2006).

**Figure 1:**
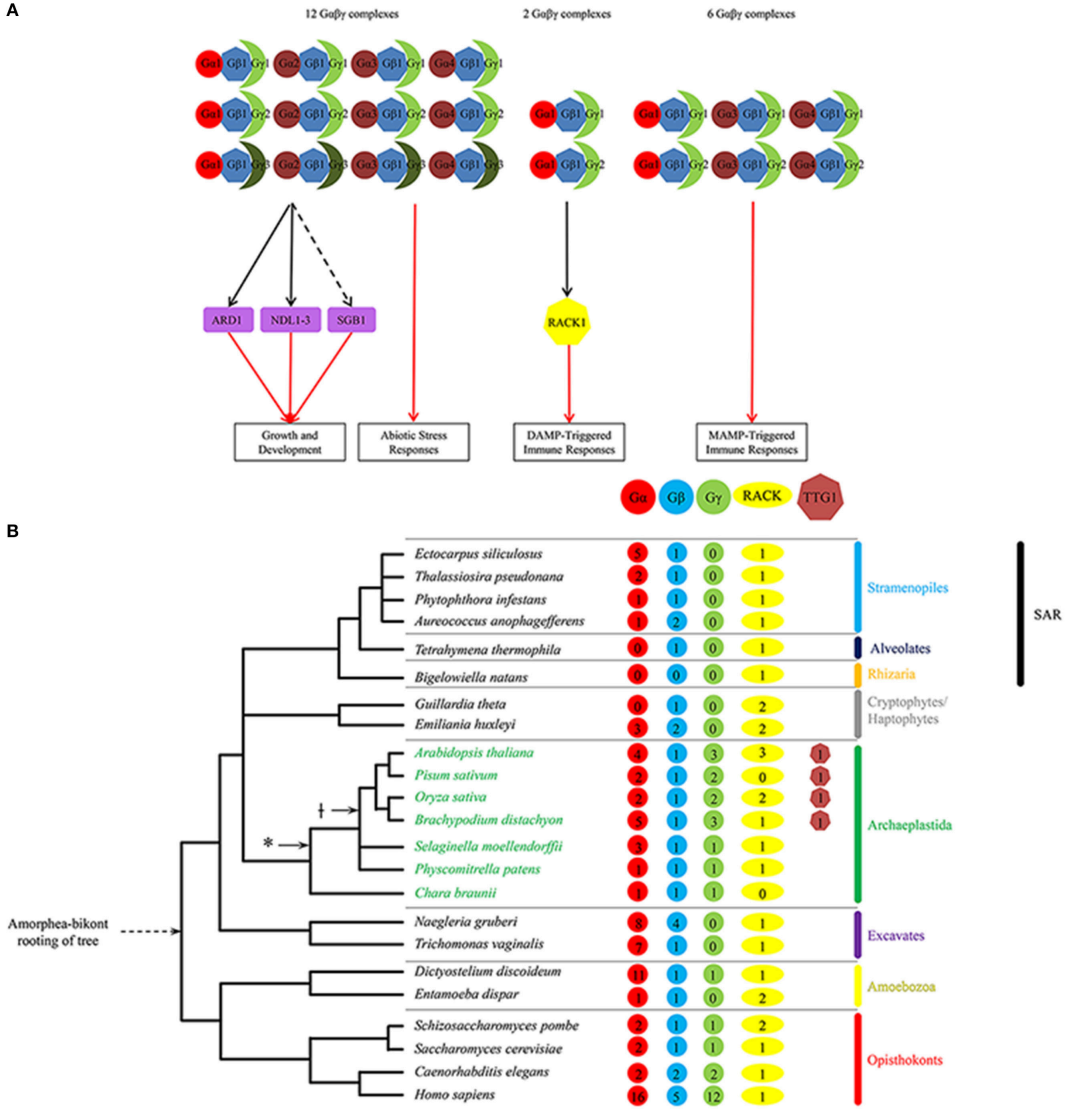
**(A)** Regulatory network of known Gβ-dependent pathways in *Arabidopsis* illustrating the interactions between G-protein subunits and between Gβ1 and its effectors for various regulated plant processes. Growth and development processes include stomatal density and opening, seed germination, hypocotyl elongation, and organ (i.e., leaf, silique, seed) morphology. Abiotic stress responses include salt stress, chemical-induced endoplasmic reticulum stress, and sugar stress. DAMP-triggered immune responses include MAP kinase activation and ROS generation. MAMP-triggered immune responses include aforementioned immune responses as well as modulation of the flavonoid anthocyanin pathway. Unbroken and broken black lines indicate indirect and direct interaction, respectively; red arrow indicates positive regulation. Shapes: heptagons, WDR-containing proteins Gβ and AtRACK1; circles, Gα proteins; moons, Gγ proteins; rectangles, downstream Gβγ effectors. For a given shape, different colors denote different classes of G-protein subunit isoforms or WDR-containing proteins. *Arabidopsis* proteins: Gβ1, AGB1; Gα1, GPA1; Gα2, XLG1; Gα3, XLG2; Gα4, XLG3; Gγ1, AGG1; Gγ2, AGG2; and Gγ3, AGG3. Note that the expanded diversity of the non-WDR-containing subunits in the complex likely provides functional specificity within plant innate immune signaling. Also note the paucity of identified effectors downstream of the G-protein complexes for all known regulated processes. **(B)** Tree depicting the number of Gβ, RACK1 and TTG1 sequences identified to date for representative species of the five eukaryote supergroups as well as two major plant evolutionary milestones: plant colonization of land (^*^) and the first appearance of the bHLH interaction motif present on constituent MYBs in the MYB-bHLH-TTG1 complex. Note that no Gβ or TTG1 sequences were identified in the Rhizaria group.

**Figure 2:**
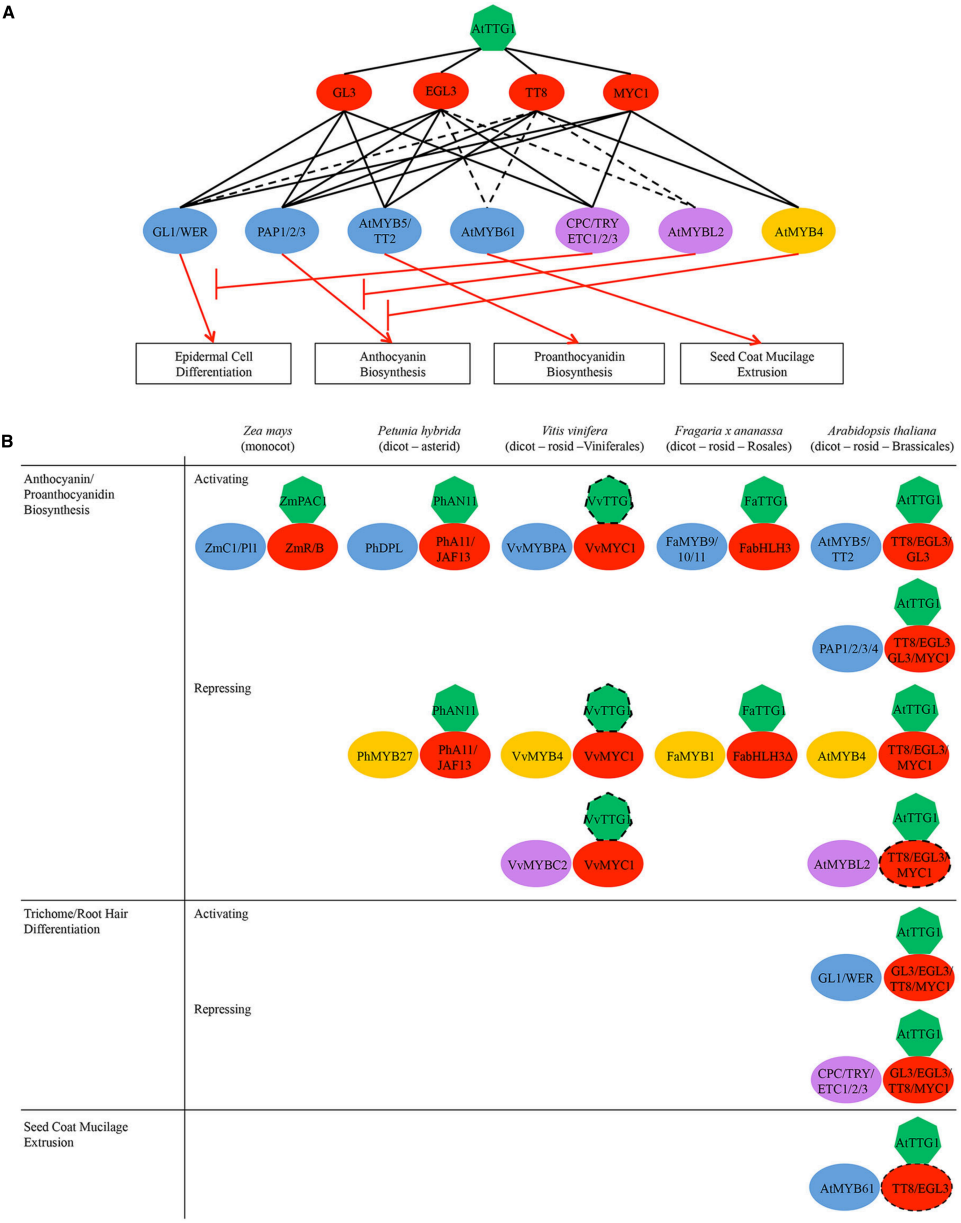
**(A)** Regulatory network of AtTTG1-dependent pathways in *Arabidopsis* illustrating the potential/indirect (broken black lines) and demonstrated (unbroken black lines) interactions among proteins/genes. Boxed are regulated processes. Key: green heptagons, WDR-containing protein AtTTG1; red ovals, bHLH transcription factors from subgroup IIIf; blue ovals, activator-type R2R3-type MYB subfamilies; yellow ovals, repressor-type R2R3-type MYBs; purple ovals, repressor-type R3-type MYB subfamilies. Note the narrowing of regulatory specificity across the tiers from top to bottom. **(B)** Schematic representation of MYB-bHLH-TTG1 (MBW) complexes regulating flavonoid defense metabolism and other processes in representative plant species of major clades within flowering plants (monocot vs. dicot, asterid vs. rosid, different rosid orders). Dotted lines indicate potential genetic/physical interactions. Note the functional conservation of MBW complexes in the regulation of flavonoid biosynthesis across different plant species.

Plant immunity, in particular, boasts two distinct but structurally similar classes of WDR-containing proteins: Gβ, and TRANSPARENT TESTA GLABRA1 (TTG1). The Gβ and TTG1 proteins are constituents of ternary regulatory complexes (Figures 1A, 2A). While the Gβ is widely conserved across a diversity of eukaryotes (Adams et al., 2011; Bradford et al., 2013), TTG1 is only present in higher plants (Figure 1B). The Gβ and TTG1 proteins are coupled to type-I membrane receptors and transcription factors, respectively, in a plant innate immune signaling pathway that convert extracellular signals into a subset of intracellular chemical defense responses (see below).

### Structural conservation of WDR-containing proteins Gβ and TTG1

**Figure 3:**
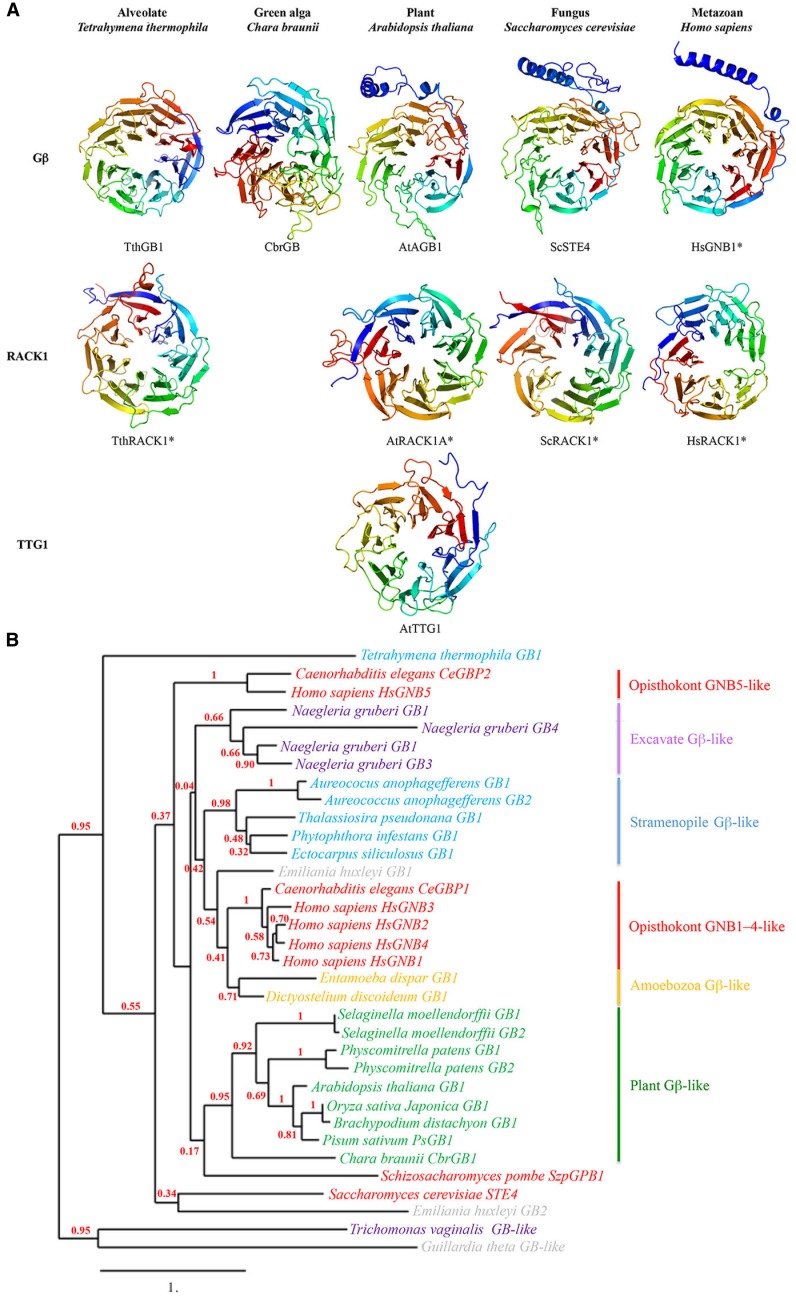
**(A)** Seven-bladed propeller-like structures of WDR-containing proteins Gβ (top row), RACK1 (middle row) and TTG1 (bottom row) proteins from an alveolate, green algal, plant, fungal, and metazoan species. Homology models were based on known structures of TthRACK1, AtRACK1A, ScRACK1, HsGNB1, and HsRACK1 (marked by asterisks) as well as predicted structures from multiple sequence templates using the PHYRE2 protein fold recognition server (www.sbg.bio.ic.ac.uk/phyre2/; Kelley and Sternberg, 2009). Acronyms: Tth, *Tetrahymena thermophila*; Cbr, *Chara braunii*; At, *Arabidopsis thaliana*; Sc, *Saccharomyces cerevisiae*; Hs, *Homo sapiens*. Note the presence of an *N*-terminal alpha helix on the plant, fungal and metazoan Gβ proteins but not on the alveolate and green algal Gβ proteins. **(B)** Maximum likelihood phylogenetic tree of Gβ sequences from representative species in the five eukaryotic supergroups. Tree was generated using MUSCLE multiple sequence alignment, PhyML phylogeny, and TreeDyn tree viewer programs (http://phylogeny.lirmm.fr; Dereeper et al., 2008). Bootstrap value (*n* = 100 replicate trees) is shown in red at the nodes. Note that the plant Gβ sequences cluster as a well-supported monophyletic group.

The common and defining structural feature of WDR-containing proteins is the seven-tandem WDR motif sequence, which adopts a seven-bladed β-propeller-like structure with three potential surfaces for molecular interactions—the top, bottom and circumference (Figure 3A; Lambright et al., 1996; Smith et al., 1999; Ullah et al., 2008; Adams et al., 2011; Ruiz Carrillo et al., 2012). Each blade of the propeller-like structure consists of four antiparallel β strands; the first three strands of one blade and the fourth strand of the next are formed by a single WDR motif; the overlap between two adjacent propeller blades provides an interlocking architecture that holds the propeller-like structure in a closed, rigid ring structure (Smith et al., 1999; Adams et al., 2011). The seven-bladed β-propeller structure is best demonstrated for the WDR-containing receptor for activated C kinase 1 (RACK1) protein, for which there are four known structures from a diversity of eukaryotes (Figure 3A; Ullah et al., 2008; Coyle et al., 2009; Rabl et al., 2011; Ruiz Carrillo et al., 2012).

Unlike TTG1, the Gβ protein additionally contains an *N*- terminal α-helix (Figure 3A) that forms a coiled-coil structure with the Gγ protein, as indicated by the crystal structure of the human Gβ HsGNB1 partially encircled by the Gα HsGNAT1 (Sondek et al., 1996). However, HsGNB1 remains the sole Gβ with a solved crystal structure, which serves as the foundation (along with a handful of solved RACK1 structures) for the predicted Gβ structures generated by structural bioinformatics. Within these confines, there is some evidence that the Gβ-specific structure mediating the Gβγ interaction may not be conserved across eukaryotes. For example, Gβ proteins from more primitive eukaryotes (e.g., alveolate *Tetrahymena thermophila* Gβ and the green alga *Chara braunii* Gβ) are predicted to lack the *N*-terminal helix (Figure 3A) but still retain the Gβγ interaction (Hackenberg et al., 2013), presumably through a novel Gβγ interaction domain(s) within the β-propeller structure. Additional crystal structures of non-metazoan Gβ sequences are needed to provide structural details on the Gβγ interaction across eukaryotes.

## Heterotrimeric G-protein complex

The most extensively studied WDR-containing protein to date is the Gβ subunit of the heterotrimeric G-protein complex, which is one of the most conserved and elaborate receptor- effector signaling mechanisms in eukaryotes. The Gβ reversibly interacts with the GDP-bound Gα subunit and forms an obligate heterodimer (Gβγ) with the Gγ subunit. While the interaction between the Gα and the Gβγ dimer serves as a molecular switch, the Gβ serves as a scaffold for effector proteins (Figure 1A). In animals and fungi, ligand perception by the heptahelical membrane receptors, G-protein-coupled receptors (GPCRs), leads to replacement of GDP with GTP in the Gα subunit and activation of the heterotrimer (Li et al., 2007; Oldham and Hamm, 2008). Upon activation, the GTP-bound Gα and Gβγ dimer dissociate from each other and from the receptor complex, releasing their bound effectors to activate various signaling cascades. Signaling terminates when the intrinsic GTPase activity of the Gα hydrolyzes GTP to GDP and the inactive heterotrimer reforms at the receptor.

### Elusive receptor-effector signaling mechanism

Although signal transduction through a heterotrimeric G-protein complex is common to animals and plants, there are some mechanistic differences between the evolutionary branches. For example, in plants and basal eukaryotes, the canonical Gα subunit isoform is self-activating, and thus does not require GPCR-like proteins for its activation (Jones et al., 2011a,b; Bradford et al., 2013). Plants also contain non-canonical Gα subunit isoforms, which have a slower rate of GTP hydrolysis (Heo et al., 2012), but it is not yet known whether they are also self-activating. In addition, canonical GPCR-like sequences are absent or rare in plants (Urano et al., 2013; Taddese et al., 2014). Instead, plants have several families of non-canonical GPCR-like sequences, three of which (GCR1, GTG1, and GTG2) have been shown to interact *in planta* with the *Arabidopsis* canonical Gα GPA1 and modulate an ABA-mediated drought response (Pandey and Assmann, 2004; Pandey et al., 2009). It remains controversial whether the GPCR-like proteins are *bona fide* GPCRs, although a recent structural bioinformatics study has found GCR1 to be a strong GPCR candidate based on its predicted heptahelical scaffold and GPCR fold (Taddese et al., 2014). Plants also contain hundreds of membrane RLP and RLK sequences (Shiu and Bleecker, 2001, 2003; Fritz-Laylin et al., 2005), two of which (the maize RLP FEA2 and the *Arabidopsis* RLK RPK2) have been shown to interact *in planta* with the maize canonical Gα CT2 and *Arabidopsis* canonical Gβ AGB1, respectively, to regulate stem cell proliferation (meristem organization; Bommert et al., 2013; Ishida et al., 2014). Finally, while all three constituents of the mammalian G-protein complex interact with the GPCR (Taylor et al., 1994, 1996), only the Gα and Gγ subunits of the *Arabidopsis* heterotrimer have been shown to interact with the receptor complex (Aranda-Sicilia et al., 2015). In the absence of receptor interaction, it remains unclear how the Gβ subunit participates in the receptor signaling complex.

Downstream, the plant Gβγ dimer has been shown to regulate the MAPK cascade by interacting directly with a MAPK protein (Bhardwaj et al., 2011; Xu et al., 2015) or by recruiting RACK1 proteins as MAPK cascade scaffolds (Cheng et al., 2015). By contrast, mammalian and fungal Gβγ dimers instead recruit the MAPK scaffolding proteins β-arrestin2 and Ste5, respectively (Witzel et al., 2012), while mammalian RACK1 proteins serve as Jun N-terminal kinase (JNK) MAPK cascade scaffolds for the protein kinase C (PKC) signaling pathway (Ron et al., 1994; López-Bergami et al., 2005). Although RACK1 is highly conserved between plants and animals (Figure 1B), β-arrestin2, Ste5 and second-messenger-regulated PKC proteins are absent in plants (Stone and Walker, 1995; Witzel et al., 2012). Despite the diversity of MAPK cascade scaffolds between plants and animals, the use of scaffolding proteins in signal transduction pathways appears universal.

### Evolutionary history of the plant Gαβγ trimer

Gβ sequences (and those of the other two G-protein subunits) are present in the genomes of all five eukaryotic supergroups Archaeplastida, Excavata, Opisthokonta, Amoebozoa, and Stramenopila/Alveolata/Rhizaria (or SAR), and are absent only in the Rhizaria subgroup of SAR (Figure 1B). Although each supergroup consists of a diversity of eukaryotes, most of which are microbial (e.g., protists and algae; Keeling et al., 2005; Burki, 2014), the best-characterized Gβ sequences are from animals/metazoans and fungi in the Opisthokonta supergroup. The oldest extant Gβ sequence in the Archaeplastida supergroup (e.g., land plants and green/red algae) is a single-copy gene found in the green alga *Chara braunii* (Hackenberg et al., 2013; Figure 1B). This green algal Gβ sequence is not distinct from the Gβ sequences present in the genomes of basal plant lineages (e.g., bryophytes and lycophytes) and the diploid genomes of higher plant lineages (Figure 3B; Urano et al., 2013), indicating that they descended from a single ancestral plant Gβ sequence. In contrast, phylogenetic analysis of metazoan Gβ sequences identified three distinct Gβ classes (GNB1–4-like, GNB5-like, and Gbe-like); the first two are found in humans, and the third is specific for arthropods (de Mendoza et al., 2014; Krishnan et al., 2015). GNB1–4-like and GNB5-like sequences are likely present in the last common metazoan ancestor and are confined within metazoans (de Mendoza et al., 2014; Krishnan et al., 2015; Figure 3B).

Previous phylogenetic analysis for ancestral plant Gβ sequences suggested that plant Gβ sequences are more closely related to Gβ sequences from the SAR (e.g., diatom) and Amoebozoa (e.g., entamoeba) supergroups than those of Excavata (Friedman et al., 2009). Although it is still not clear how the eukaryotic supergroups relate to one another, the most popular hypothesis (Amorphea-bikont rooting) places the root of the eukaryotic tree between the last common ancestor of the amoebozoans and opisthokonts and the remaining eukaryotes (Keeling et al., 2005; Burki, 2014). The Amorphea-bikont rooting positions the Gβ sequences in the Excavata supergroup between the plant Gβ sequences and those of the amoebozoans and opisthokonts (Figure 1B). Phylogenetic analysis of a representative sampling of Gβ sequences from all five supergroups supports this hypothesis by sandwiching the Gβ sequences in the Excavata supergroup between the plant Gβ sequences and the animal GNB1–4-like sequences (Figure 3B).

### Combinatorial diversity of plant G-proteins

Although the heterotrimeric G-protein complex consists of three subunits, subunit isoforms can give rise to many heterotrimeric combinations, limited in part by amino acid sequence differences in the contact regions that lead to selective interactions. Given the large number of known G-protein-mediated signaling pathways, a diversity of G-protein isoforms is needed for signaling specificity (Wettschureck and Offermanns, 2005). For example, the human genome encodes 16 Gα, 5 Gβ, and 12 Gγ subunit isoforms, allowing for approximately 700 potential Gαβγ combinations (Hillenbrand et al., 2015; Figure 1B). By contrast, *Arabidopsis thaliana*, like most diploid plants, contains 4 Gα (GPA1, XLG1–3), one Gβ (AGB1), and 3 Gγ (AGG1–3) subunit isoforms, allowing for 12 potential Gαβγ combinations (Figure 1; Ma et al., 1990; Weiss et al., 1994; Mason and Botella, 2000, 2001; Zhu et al., 2009; Thung et al., 2012; Chakravorty et al., 2015; Maruta et al., 2015). This number falls short of the specificity needed for the large number of known G-protein-mediated signaling pathways regulating fundamental processes in plants, and remains a bottleneck issue in plant G-protein signaling (Urano et al., 2013).

The sole Gβ subunit isoform is a limiting factor for plant G-protein combinatorial diversity. There are different complex models on how one Gβ subunit isoform is able to transduce so many diverse signals (Urano and Jones, 2014). In addition, the ubiquitous presence of Gβ-like sequences across plant genomes has led to a hypothesis on the existence of additional non- canonical classes of plant Gβ subunits that have yet to be discovered, a situation analogous to the recent discoveries of new classes of plant Gα sequences (XLG1–3-like) and plant Gγ subunits (AGG3-like; Lee and Assmann, 1999; Thung et al., 2012). The XLG1–3-like Gα subunit differs from the canonical Gα subunit in its possession of a long N-terminal extension of unknown function and its nuclear- and plasma membrane- localization (Ding et al., 2008; Chakravorty et al., 2015; Maruta et al., 2015). Similarly, the AGG3-like Gγ subunit differs from the canonical Gγ subunit in its possession of a C-terminal extension that is cysteine-rich and of unknown function (Chakravorty et al., 2011; Trusov et al., 2012).

Aside from genetic interaction data, there is physical interaction evidence from yeast three-hybrid studies supporting interaction specificity within the heterotrimer and its putative coupled receptor/adaptor. For example, the *Arabidopsis* Gα subunit isoforms, XLG1 and XLG2 have been shown to strongly interact with the Gβγ heterodimers AGB1-AGG1/2, while the Gα subunit isoform GPA1 strongly interacts with the Gβγ heterodimer AGB1-AGG3 and XLG3 strongly interacts with all three Gβγ heterodimers AGB1-AGG1/2/3 (Chakravorty et al., 2015; Maruta et al., 2015), suggesting that all three Gγ isoforms are somewhat selective of their interaction partners, each preferring two of the four Gα isoforms. In addition, yeast split-ubiquitin and Bimolecular Fluorescence Complementation (BIFC) studies indicate that the other two Gγ isoforms AGG1/2 mediate the interaction between the plant heterotrimer and the co-receptor proteins BAK1 and CERK1 (Aranda-Sicilia et al., 2015). These reports are consistent with similar reports of animal Gγ isoforms conferring specificity to the G-protein complex-GPCR interaction (Im et al., 1988; Kisselev and Gautam, 1993).

### G-protein complexes in defense

One of the best-characterized functions of the *Arabidopsis* heterotrimeric G-protein complex is in plant innate immunity, where it participates in multiple immune signaling pathways and defense responses (e.g., reactive oxygen species (ROS) production, mitogen-activated protein kinase (MAPK) activation, defense gene activation, callose deposition, and programmed cell death) against a variety of fungal (Llorente et al., 2005; Trusov et al., 2006, 2007, 2009; Delgado-Cerezo et al., 2012; Torres et al., 2013) and bacterial pathogens (Zhang et al., 2008; Ishikawa, 2009; Zeng and He, 2010; Lee et al., 2013; Liu et al., 2013; Torres et al., 2013). Evidence of a physical interaction between a plant heterotrimer and a ligand-binding innate immune receptor (e.g., FLS2, EFR, LYK4/5) is still elusive, although a recent report showed a direct interaction between the canonical Gα GPA1 and the Gγ isoforms AGG1/2 (but not the Gβ AGB1) with the co-receptor proteins BAK1 and CERK1 by yeast split-ubiquitin assay and BiFC studies (Aranda-Sicilia et al., 2015). If validated, this is the first report of a novel plant-specific interaction between a heterotrimer and a receptor complex via co-receptor adaptors. If the plant heterotrimer is coupled directly to the receptor complex, then further research is needed to understand how the plant heterotrimer converts MAMP and/or DAMP signals from the receptors into intracellular defense responses, especially if the heterotrimer is self-activating.

Nearly all of the *Arabidopsis* G-protein subunit isoforms (save two—XLG1 and AGG3) participate in plant defense (Figure 1A; Maruta et al., 2015) and an even smaller subset of G- protein subunit isoforms in a bacterial DAMP-triggered immune pathway involving RACK1 proteins as MAPK cascade scaffolds (Figure 1A; Cheng et al., 2015). The sole Gβ AGB1 participates in all G-protein-mediated processes, and positively contribute to all tested immune responses, including ROS production, callose deposition, MAPK activation, defense gene activation and programmed cell death (Llorente et al., 2005; Maeda et al., 2009; Liu et al., 2013; Torres et al., 2013). Among the Gα subunit isoforms, XLG2 is the major contributor to resistance against the hemibiotrophic bacterium *Pseudomonas syringae*, necrotrophic fungi *Alternaria brassicicola* and *Plectosphaerella cucumerina*, and the hemibiotrophic fungus *Fusarium oxysporum*. The loss-of-function *xlg2* mutant most closely recapitulates the phenotypes of the loss-of-function *agb1* mutant in its pathogen susceptibility (Llorente et al., 2005; Trusov et al., 2006; Zhu et al., 2009; Torres et al., 2013; Maruta et al., 2015), and the XLG2 protein was shown to interact with the AGB1 protein *in planta* by co-immunoprecipitation of overexpressed proteins (Zhu et al., 2009). In addition, the Gα isoform GPA1 contributes to bacterial resistance by mediating stomatal closure, a MAMP- triggered immune response that retards pathogen entry through the stomata (natural openings in the plant surface; Zhang et al., 2008), while the Gα isoform XLG3 contributes partly to resistance against *Fusarium oxysporum* (Maruta et al., 2015) through an unknown mechanism. Among the Gγ subunit isoforms, AGG1 and AGG2 are mostly redundant in their contribution to plant immunity (Trusov et al., 2007; Thung et al., 2013). The loss-of-function *agg1/agg2* double mutant recapitulates the phenotypes of the *agb1* mutant in pathogen susceptibility (Trusov et al., 2007; Liu et al., 2013; Torres et al., 2013; Maruta et al., 2015). These results suggest that multiple G-protein combinations are required for a successful plant defense response against a majority of pathogens, with the Gβ playing a central but undefined role in immune signaling.

### Search for G-protein complexes in pathogenesis

Although compatibility between plants and their pathogens leads to disease and symptom development, it is rarely found in nature due to the effectiveness of the plant innate immune system. One exceptional case is the small family of GPCR-like mildew resistance locus O (MLO) receptor proteins, which are found throughout flowering plants. A subset of MLO proteins has been shown to be a conserved requirement for the compatibility of monocots and dicots with their adapted powdery mildew pathogens (Devoto et al., 2003; Consonni et al., 2006; Humphry et al., 2006). While it remains controversial whether GPCR-like proteins are *bona fide* GPCRs, some *Arabidopsis* MLO proteins have predicted heptahelical scaffolds, GPCR folds, and G-protein coupling (Taddese et al., 2014). However, the three MLO proteins involved in fungal pathogenesis (AtMLO2/6/12) do not contain these GPCR hallmarks and thus are unlikely to function as canonical GPCRs (Taddese et al., 2014). In addition, attempts to couple the two plant heterotrimers (GPA1-AGB1-AGG1/2) to the MLO receptor were not successful (Lorek et al., 2013). Still, further research is needed to discern whether other heterotrimeric combinations are involved in regulating the compatibility between plants and their pathogens.

## Ternary MYB-bHLH-TTG1 complexes

### Flavonoids in defense

**Figure 4:**
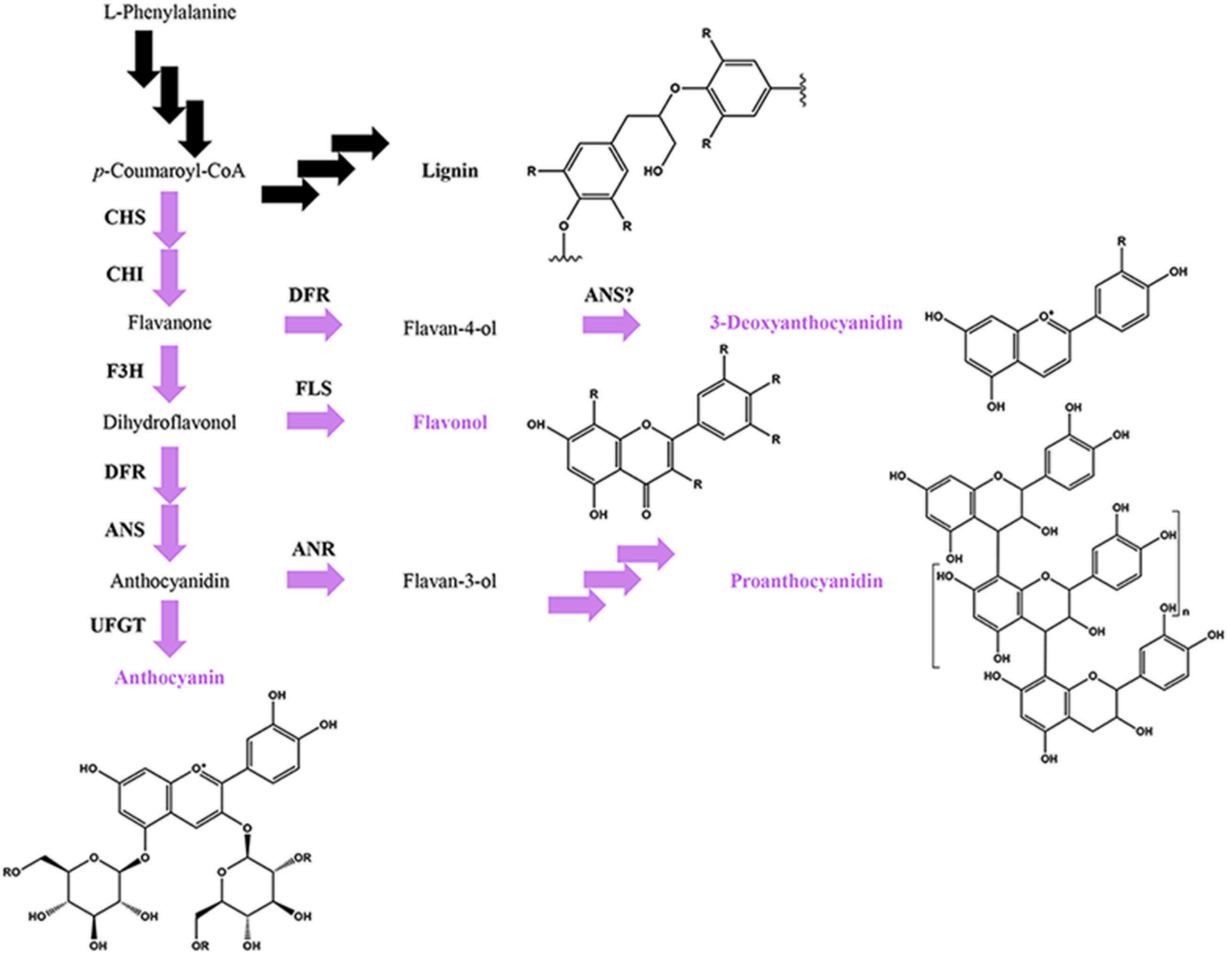
**Simplified phenylpropanoid pathway depicting the flavonoid (in purple) and lignin branch pathways, major flavonoid classes and basic flavonoid structures**. Multiple arrows indicate multiple enzymatic steps. Key flavonoid steps: CHS, chalcone synthase; CHI, chalcone isomerase; F3H, flavanone 3-hydroxylase; FLS, flavonol synthase, DFR, dihydroflavonol 4-reductase; ANS, anthocyanidin synthase; ANR, anthocyanidin reductase; UFGT, UDP-glucose flavonoid 3-*O*-glucosyltransferase.

Plant secondary (or defense) metabolites are essential for plant survival in complex environments, and are the primary means of defense against microbial pathogens as well as insect herbivores and competitor plants (Dixon, 2001; Kliebenstein, 2013). The phenylpropanoids represent one of the largest and most ancient families of defense metabolites in land plants. The two largest subfamilies within the phenylpropanoids are the flavonoids and the lignins, whose partially overlapping, competing branch pathways share the first three steps in the phenylpropanoid biosynthetic pathway (Figure 4). Among the flavonoid classes, the anthocyanins, proanthocyanidins and 3-deoxyanthocyanidins have been shown to be critical for disease resistance in a number of plant species. For example, the 3-deoxyanthocyanidin phytoalexins in *Sorghum* contribute to localized resistance against the *Sorghum* anthracnose disease fungus *Colletotrichum sublineolum*, the southern leaf blight fungus *Cochliobolus heterostrophus* and head smut fungus *Sporisorium reilianum* (Nicholson et al., 1987; Snyder and Nicholson, 1990; Lo et al., 1999; Zuther et al., 2012). In grapevine, proanthocyanidins (also known as condensed tannins) contribute to resistance against the gray mold fungus *Botrytis cinerea* by competitively inhibiting fungal laccases involved in plant cell wall degradation and phytoalexin detoxification (Pezet et al., 1992; van Baarlen et al., 2007). In *Arabidopsis*, the colorful anthocyanins may be the molecular basis of the ISR that is induced by the biocontrol fungus *Trichoderma hamatum* against *Botrytis cinerea* (Mathys et al., 2012; Kottb et al., 2015). Additionally, the *Trichoderma*-synthesized volatile 6-pentyl-α-pyrone may be the MAMP signal responsible for triggering ISR (Kottb et al., 2015). Although the identities of the flavonoid metabolite(s) involved in ISR remain unknown, mutant analysis confirmed that flavonoid biosynthesis is required for ISR (Mathys et al., 2012).

Anthocyanins, proanthocyanidins and 3-deoxyanthocyanidins represent partially overlapping, competing pathways within the flavonoid biosynthetic pathway (Figure 4; Hipskind et al., 1996; Winkel-Shirley, 2001). In addition, the flavonoid pathway itself competes with the lignin pathway for shared resources (Figure 4). The immediate biochemical and physiological needs for the defense response are best illustrated during the *de novo* synthesis of defense metabolites critical to disease resistance, where the synthesis of more common/less essential phenylpropanoids are repressed in order to maintain metabolic balance between the competing pathways (Kombrink and Hahlbrock, 1990). For example, in *Arabidopsis*, the bacterial MAMP flg22 transcriptionally upregulates the synthesis of lignins, while repressing the light-dependent production of anthocyanins (Saijo et al., 2009; Adams-Phillips et al., 2010; Schenke et al., 2011). Similarly, in *Sorghum*, unknown fungal-derived signals transcriptionally upregulate the synthesis of the 3-deoxyanthocyanidins, while repressing light-dependent production of anthocyanins (Weiergang et al., 1996; Lo and Nicholson, 1998; Wharton and Nicholson, 2000; Shih et al., 2006). Defense metabolites can also be synthesized from two or more overlapping pathways, requiring more complex pathway regulation. For example, most monocots, including maize, rice, and wheat, incorporate the flavonoid tricin into the lignin polymer (Lan et al., 2015), although it is not yet known whether the flavonoid content contributes to the defense function of lignins.

### Elusive transcription factor-regulon mechanism

In plants, the majority of the non-homologous gene constituents of well-characterized multi-gene pathways in defense metabolism and cell differentiation are not genetically clustered as they are in fungi and bacteria, but are distributed throughout the plant genome (Lappin et al., 2006; Kliebenstein and Osbourn, 2012). Instead, to facilitate their common regulation, co-regulated genes along a pathway or sections within typically share common or overlapping sets of promoter elements that are bound by specific transcription factor, and thus are transcriptionally clustered into an operon- like gene module known as a regulon. This functional gene organization allows for a limited set of transcriptional regulators to combinatorially control a regulon for pathway function. The best-studied transcriptional regulator of pathways in defense metabolism in higher plants is the MYB-bHLH-WDR (MBW) complex, wherein the sole constituent WDR-containing protein is known as TTG1 and the MYB and bHLH constituents are paralogous transcription factors from small subfamilies (analogous to classes of G-protein subunit isoforms; Figure 2A). MYB constituents of the MBW complexes are either R2R3-type or R3-type MYBs, where R stands for the number of adjacent repeats of the MYB DNA-binding, helix-turn-helix domain; plant MYBs lack the first repeat (Kranz et al., 1998; Stracke et al., 2001; Dubos et al., 2010).

The pathway specificity of the MBW complex is determined by the diversity of the MYB constituent, limited in part by the overlapping functions of paralogous members within a MYB subfamily (Stracke et al., 2001). However, *in vitro* promoter binding studies to date indicate that R2R3-type MYBs from different subfamilies appear to bind to the same or very similar *cis*-regulatory elements, which are described generally as “AC-like” sequences (e.g., TACC(T/A)A(C/A)C (MBSIIG1–4 motif), CACC(T/A)A(C/A)C (MBSIIG5–8 motif), ACCTACC (AC-I or SMRE-8 motif), ACCAACC (AC-II or SMRE-4 motif), ACCTAAC (AC-III or SMRE-7 motif), ACC(T/A)ACC (AC-I/SMRE-8 or AC-II/SMRE-4 motif), and ACCCGCC) (Grotewold et al., 1994; Williams and Grotewold, 1997; Romero et al., 1998; Zhao et al., 2007; Zhou et al., 2009; Fornalé et al., 2010; Prouse and Campbell, 2013). However, *in vitro*-defined consensus motifs may not be sufficient and/or present in the majority of their *in vivo* DNA target sequences, as was demonstrated for several animal transcription factors (Carr and Biggin, 1999; Yang et al., 2006; Rabinovich et al., 2008) and the maize R2R3-type MYB ZmMYBP1 from subgroup 7, which was found to preferentially bind to sequences containing 6 to 8 repeats of CxxC (where X corresponds to any nucleotide) with no preference for A or T between the C base pairs (Morohashi et al., 2012). Currently, it remains unclear how the different MYB subfamilies confer pathway specificity to the MBW complex.

Because AC-like sequences are also typically present in the promoters of activator- and repressor-type R2R3-type MYB genes, constituent MYBs not only participate in MBW complexes but also can transcriptionally regulate other components in the complexes and/or competing MYBs via autoregulatory, feedback or feedforward loops (Baudry et al., 2006; Zhao et al., 2007). In addition, the expression of repressor-type MYBs is typically development-specific or stress-responsive and thus can serve to fine-tune pathway regulation (Jin et al., 2000; Preston et al., 2004; Fornalé et al., 2014). For example, chromatin immunoprecipitation experiments in *Arabidopsis* have shown that the WDR-containing TTG1 and the bHLH constituent GL3 are associated with the promoters of pathway genes in anthocyanin and proanthocyanidin metabolism as well as the promoters of repressor- and R3-type MYBs CPC, TRY, and ETC1 (Morohashi et al., 2007; Zhao et al., 2008).

### Role of TTG1 in defense

The WDR-containing TTG1 has both direct and indirect roles in plant defense. For example, heterologous expression of the *Sorghum* R2R3-type MYB SbY1 in maize induced 3-deoxyanthocyanidin synthesis and enhanced resistance against leaf blights (Ibraheem et al., 2010, 2015). Although maize contains the SbY1 ortholog ZmP1 (Grotewold et al., 1994) and the necessary flavonoid biosynthetic genes, ZmP1 does not participate in MBW complexes nor induce 3-deoxyanthocyanidin synthesis in response to fungal attack (Ibraheem et al., 2015). These observations suggest that SbY1 requires additional regulatory factors to induce 3-deoxyanthocyanidin synthesis in *Sorghum* (and in maize). One of these factors may be the *Sorghum* TTG1 ortholog Tan1 (Mizuno et al., 2014). QTL analysis identified *Tan1* gene as a major determinant of the relative proportions of 3-deoxyanthocyanidins responsible for the pathogen-induced color variation in *Sorghum* (Mizuno et al., 2014). TTG1 has also been found to play a direct role in plant defense in dicots. For example, in tobacco, TTG1 physically interacts with the oomycete-specific effector ParA1 to activate plant immune responses, such as ROS generation and programmed cell death (Wang et al., 2009).

### Combinatorial MYB-bHLH interactions

TTG1 serves as a scaffold for the combinatorial interactions between the MYB and bHLH constituent isoforms, which are essential for regulating the binding specificity and transcriptional activity of the MBW complex. Both the MYB and bHLH constituents can be sequestered by other bHLH and MYB proteins, respectively, to form non-functional MYB-bHLH complexes and thus disrupt the MBW complex (Burr et al., 1996; Sawa, 2002; Esch et al., 2003; Kirik et al., 2004; Simon et al., 2007; Tominaga et al., 2007; Dubos et al., 2008; Matsui et al., 2008). In addition, the bHLH constituent can form functional homo- or heterodimers with other bHLH proteins through their hydrophobic helix-loop-helix regions and/or ACT domains (Feller et al., 2006; Kong et al., 2012), whereas the MYB constituent is functionally dependent on their its bHLH partners (Lee and Schiefelbein, 1999; Zhang et al., 2003; Hernandez et al., 2007).

The MYB constituent determines the transcriptional activity of the complex (i.e., activating or repressing). There are two repressor-type MYB subfamilies: R2R3-type MYBs from subgroup 4 (e.g., *Arabidopsis* AtMYB4, petunia PhMYB27, grapevine VvMYBC2-L1–3) and a single group of R3-type MYBs (e.g., *Arabidopsis* CPC, TRY, ETC1, AtMYBL2). Members of both subfamilies contain bHLH interaction motifs as well as one or more repressor motifs, including the ethylene response factor (ERF)-associated amphiphilic repressor (EAR) motif LNLxL (Kranz et al., 1998; Jin et al., 2000; Cavallini et al., 2015). Genetic and *in vitro* physical interaction studies in *Arabidopsis*, petunia and grapevine suggest that MBW complexes may be able to switch between activating and repressive modes through a modular exchange of MYB components between activator- and repressor-type MYB subfamilies (Jin et al., 2000; Zimmermann et al., 2004; Albert et al., 2014; Cavallini et al., 2015). It remains to be seen whether repressor-type MBW complexes exist *in planta*.

### Evolutionary history of the MYB-bHLH-TTG1 complex

Not all MYB and bHLH subfamilies participate in MBW complexes. To date, only bHLHs from subgroup IIIf and MYBs containing the bHLH interaction motif [DE]Lx_2_[RK]x_3_Lx_6_Lx_3_R (Zimmermann et al., 2004) have been shown to directly interact with TTG1 and participate in MBW complexes (Payne et al., 2000; Stracke et al., 2001; Heim et al., 2003; Zhang et al., 2003; Baudry et al., 2004; Zimmermann et al., 2004). bHLHs from subgroup IIIf are the best-studied bHLH proteins in plants and function only in complex with R2R3-type MYBs from subgroups 4, 5, 6, and 15 as well as R3-type MYBs. Although the bHLH interaction motif arose early in land plant evolution and is conserved between angiosperms and gymnosperms (Figure 1B; Bedon et al., 2007), the bHLHs from subgroup IIIf themselves are evolutionarily older, arising before the origin of the mosses approximately 400 million years ago (Carretero-Paulet et al., 2010; Pires and Dolan, 2010). In contrast, TTG1 sequences are present in angiosperms, but not in gymnosperms or more ancient plant lineages (Figure 1B; Humphries et al., 2005; Brueggemann et al., 2010; Ben-Simhon et al., 2011). This suggests that the bHLHs from subgroup IIIf functioned as dimers in transcriptional regulation long before the MBW complex, and that ancestral MBW complexes may have originated as MYB-bHLH dimers that then recruited the WDR-containing protein TTG1 to aid in complex stability and/or subnuclear localization (Zhao et al., 2008).

The role of MBW complexes in mediating the synthesis of anthocyanins and proanthocyanidins appears to be conserved in diverse species of flowering plants, including *Arabidopsis* and related species, *Medicago*, strawberry, petunia and maize (Figure 2B; Grotewold et al., 2000; Carey et al., 2004; Dressel and Hemleben, 2009; Pang et al., 2009; Zhang et al., 2009; Schaart et al., 2013; Albert et al., 2014; Chopra et al., 2014). For example, maize uses the MBW complexes ZmC1/PL1-ZmR/B-ZmPAC1 to regulate the anthocyanin pathway (Goff et al., 1990; Tuerck and Fromm, 1994; Selinger and Chandler, 1999; Walker et al., 1999; Carey et al., 2004). In addition, *Arabidopsis* and other dicots have evolved two different classes of MBW complexes to separately regulate the anthocyanin and proanthocyanidin pathways (Figure 2A). *Arabidopsis* MBW complexes PAP1/2/3- EGL3/GL3/TT8-AtTTG1 activate anthocyanin biosynthesis genes (e.g., *DFR*; Figures 2A, 4; Walker et al., 1999; Borevitz et al., 2000; Nesi et al., 2000, 2001; Zhang et al., 2003; Zimmermann et al., 2004; Teng et al., 2005; Gonzalez et al., 2008); whereas, MBW complexes AtMYB5/TT2-GL3/EGL3/TT8-AtTTG1 activate genes specific for proanthocyanidin biosynthesis (e.g., *BAN/ANR*; Figures 2A, 4). Both classes of MBW complexes share the same bHLH and WDR constituents, leaving their MYB constituents to determine the promoter target activation and pathway specificity of the MBW complex. In addition, the MYB constituents AtMYB5/TT2 from subgroup 5 and PAP1-3 from subgroup 6 are closely related. They are in fact so close that the sequence variation at the R/G^39^ motif in their R2 motifs and at the A/SNDV or DNEI^90–93^ motif in their R3 motifs (also known as the A2 box; Cavallini et al., 2015) has been shown to be sufficient to confer pathway specificity (Heppel et al., 2013). The same A2 box also confers pathway specificity for the repressor-type MYBs (Cavallini et al., 2015).

## Conclusion

Plants are the basis for human nutrition and a renewable source for fuel and chemical feedstocks. Diminishing food security from plant disease/pests, climate instability and population growth, concomitant with rising energy costs and dwindling petrochemical-based fossil fuel supplies, have placed high demands on the productivity of food crops and other crops of economic importance (Krattiger, 1997; Lobell et al., 2011; Lobell and Gourdji, 2012; UN-DESA, 2013). Because WDR-containing trimeric complexes are at the heart of immune signaling and transcriptional regulation of chemical defenses, continued basic and translational research on these complexes in plant immunity will certainly improve agriculture and food security as well as our understanding of fundamental processes of signal transduction and gene regulation.

Plant Gβ sequences are ubiquitous across all five eukaryotic supergroups, with only a handful of species having more than two Gβ sequences (Figure 1B). Its signaling mechanism has evolved very slowly and yet pervasively so that it cannot be easily extricated from multiple immune signaling pathways (Figure 1A). In contrast, TTG1 is plant-specific and arose after the functional dimerization of its MYB-bHLH partners (Figure 2A). Evolution has added several layers of specialization to its regulatory mechanism, including multiple partially redundant bHLH and MYB partners, regulatory loops, and repressor-type MYBs to fine-tune the activation of its target pathways (Figure 2B). While its regulatory mechanism has evolved more quickly, TTG1 and its complex are more dispensable and limited in reach, affecting only a handful of pathways in the most evolved plants (Figure 2B).

Despite differences in evolutionary history, mechanism and target pathways, the plant Gβ and TTG1 proteins represent the apex of the hierarchy of network interactions in their respective pathways (Figures 1A, 2A), and are the sole constituents of their respective complexes to preside over all signaling and regulatory pathways in plant immunity that are mediated by WDR-containing ternary complexes. While there are still many open questions concerning the dynamics of these complexes and the specificity of their interactions with other protein partners and their downstream effectors or target DNA sequences, the large and still-growing body of research on these proteins and their complexes underscores the importance of these signaling and regulatory complexes.

## Author contributions

JM wrote GB section. WC wrote TTG1 section. NC wrote introduction and edited manuscript. All three wrote conclusion.

### Conflict of interest statement

The authors declare that the research was conducted in the absence of any commercial or financial relationships that could be construed as a potential conflict of interest.
